# Use of 30-Hz Accelerated iTBS in Drug-Resistant Unipolar and Bipolar Depression in a Public Healthcare Setting: A Case Series

**DOI:** 10.3389/fpsyt.2021.798847

**Published:** 2022-01-12

**Authors:** Filippo Cantù, Giandomenico Schiena, Domenico Sciortino, Lorena Di Consoli, Giuseppe Delvecchio, Eleonora Maggioni, Paolo Brambilla

**Affiliations:** ^1^Department of Pathophysiology and Transplantation, University of Milan, Milan, Italy; ^2^Department of Neurosciences and Mental Health, Fondazione Istituto di Ricovero e Cura a Carattere Scientifico (IRCCS) Ca' Granda Ospedale Maggiore Policlinico, Milan, Italy

**Keywords:** iTBS, accelerated, TMS, TRD, depression, Bipolar Disorder

## Abstract

**Background:** Depressive episodes, especially when resistant to pharmacotherapy, are a hard challenge to face for clinicians and a leading cause of disability worldwide. Neuromodulation has emerged as a potential therapeutic option for treatment-resistant depression (TRD), in particular transcranial magnetic stimulation (TMS). In this article, we present a case series of six patients who received TMS with an accelerated intermittent theta-burst stimulation (iTBS) protocol in a public healthcare setting.

**Methods:** We enrolled a total number of six participants, affected by a treatment-resistant depressive episode, in either Major Depressive Disorder (MDD) or Bipolar Disorder (BD). Patients underwent an accelerated iTBS protocol, targeted to the left dorsolateral prefrontal cortex (DLPFC), 3-week-long, with a total of 6 days of overall stimulation. On each stimulation day, the participants received 3 iTBS sessions, with a 15-min pause between them. Patients were assessed by the Hamilton Rating Scale for Depression (HAM-D), the Montgomery-Asberg Depression Rating Scale (MADRS), the Hamilton Rating Scale for Anxiety (HAM-A), and the Mania Rating Scale (MRS). At baseline (T_0_), at the end of the second week (T_1_), and at the end of the cycle of stimulation (T_2_).

**Results:** The rANOVA (repeated Analysis of Variance) statistics showed no significant effect of time on the rating scale scores, with a slight decrease in MADRS scores and a very slight increase in HAM-A and HAM-D scores. No manic symptoms emerged during the entire protocol.

**Conclusions:** Although accelerated iTBS might be considered a less time-consuming strategy for TMS administration, useful in a public healthcare setting, our results in a real-word six-patient population with TRD did not show a significant effect. Further studies on wider samples are needed to fully elucidate the potential of accelerated iTBS protocols in treatment-resistant depression.

## Introduction

Depressive episodes, either in Major Depressive Disorder (MDD) or in Bipolar Disorder (BD), are recognized as leading causes of disability worldwide (1, 2), having a significant economic and social burden (3). Although numerous therapeutic approaches are currently available for depressive episodes, it is estimated that more than 30% of patients affected by MDD fail to respond to pharmacological therapy (4–6), and up to 40% of patients with bipolar depression are classified as non-responders (7, 8).

Clinicians are frequently forced to manage treatment-resistant depression (TRD), which is usually defined as the failure to respond to at least two pharmacological trials at an adequate dose for at least 6 weeks (5, 9). When a depressive episode appears in the context of a Bipolar Disorder, resistance is defined as the failure to respond to at least two separate monotherapeutic trials at an adequate dose for at least 8 weeks, or one monotherapy with one combination treatment (8).

In this scenario, neuromodulation has emerged as a potential therapeutic option. Among neuromodulation techniques, repetitive Transcranial Magnetic Stimulation (rTMS) is the most commonly employed. It induces an electrical current in a targeted region of the brain through a magnetic field generated by a coil, positioned on the scalp (10). rTMS delivers trains of stimuli at high frequencies (i.e., more than 5 Hz) or low frequencies (i.e., <5 Hz), which are thought to exert excitatory or inhibitory effects on the underlying cortex, respectively (11).

A specific way of delivering rTMS is Theta Burst Stimulation (TBS), which uses triplets of pulses (i.e., bursts) at high frequency (in the 30–50 Hz range), with an inter-burst interval of 200 ms (5 Hz). TBS can be performed using continuous (cTBS) protocols, which reduce the excitability of the underlying cerebral cortex, or with intermittent protocols (iTBS), which have enhancing properties (10, 12). Theta rhythms facilitate long-term potentiation (13–16), thus TBS protocols are thought to induce more rapid and long-lasting effects on synaptic plasticity than conventional rTMS (17). Recently, the non-inferiority of iTBS compared to excitatory rTMS has been demonstrated in treating depressive episodes in MDD (18). Moreover, TBS protocols last 3 min, conventional 10 Hz rTMS sessions 37.5 min (19). Therefore, TBS seems to be more advantageous in terms of cost-utility (18) and appears of particular interest in public healthcare settings, where these issues are crucial.

Accelerated TMS (aTMS) is another strategy to improve the conventional TMS protocols. It consists of delivering multiple sessions on the same day, to condense the overall stimulation time and accelerate the clinical response (20). To date, aTMS has shown encouraging results, as reported by a recent meta-analysis (21).

Combining the two abovementioned strategies, the use of TBS in an accelerated protocol might be an option in order to further optimize the cost-utility of rTMS (4, 22). Indeed, a recent accelerated iTBS protocol, improved by the use of neuronavigation, has shown promising results in TRD (23).

In this case-series, we presented and discussed the effectiveness of an accelerated iTBS protocol employed in a group of six real-world patients with TRD recruited in public service.

## Materials and Methods

### Participants

We enrolled a total number of six participants, affected by a depressive episode, either in MDD or in BD, diagnosed according to the Diagnostic and Statistical Manual fifth edition (DSM-5) criteria. Each patient was evaluated by a psychiatrist, who collected a detailed medical history and performed a Structured Clinical Interview for DSM, clinical version (SCID-CV) before enrolment. The patients were recruited in the outpatient services of the Psychiatry Unit of the Department of Neurosciences and Mental Health at Fondazione IRCCS Ca' Granda Ospedale Maggiore Policlinico, Milan, Italy. Participants before their enrolment signed a written informed consent.

### Inclusion and Exclusion Criteria

The inclusion criteria were: (a) age: between 21 and 70 years; (b) right-handedness; (c) current depressive episode according to DSM-5 criteria, either in MDD or in BD; (d) no pharmacological change in the last 4 weeks before the beginning of the stimulation cycle; (e) pharmacological resistance (i.e., at least two adequate trials of antidepressants in MDD); (f) at least two separate monotherapeutic trials, or one monotherapy with one combination treatment, in BD.

The exclusion criteria were: (a) diagnosis of schizophrenia or other psychotic disorders; (b) diagnosis of neurological disorders; (c) diagnosis of substance use disorder in the last 6 months, according to DSM-5 criteria; (d) contraindications to TMS according to the Consensus Recommendations for the safe and effective application of rTMS (24).

### Protocol

The study started in October 2019 and ended in October 2020. TMS stimulation was delivered using an STM9000 Magnetic Stimulator (ATES Medical Device, Italy) with a 70 mm butterfly cooled coil.

Accelerated iTBS was delivered to the left dorsolateral prefrontal cortex (DLPFC), in a 3-week protocol, with 3 days of stimulation during the first week, 2 days of stimulation during the second week, and 1 day of stimulation in the last one, for a total of 6 days of stimulation. A total of 18 stimulation sessions were performed. On each stimulation day, the participants received 3 iTBS sessions, with a 15-min pause between them. This procedure was similar to an accelerated rTMS protocol demonstrated to be as effective as the standard rTMS protocol in a recent study (20).

The site of stimulation was found 5 cm anterior to the site of motor threshold (MT) determination, in a parasagittal plane. We delivered, at 80% of the motor threshold (MT), triplet 30 Hz bursts, repeated at 5 Hz; 2 s on and 12.3 s off; 600 pulses per session, 1,800 pulses per day of stimulation. All these parameters were set according to international safety guidelines (25).

The protocol was approved by the local Ethics Committee.

### Rating Scales

Clinical evaluation was performed by means of the following scales: (a) Hamilton Rating Scale for Depression [HAM-D; (26)]; (b) Montgomery-Asberg Depression Rating Scale [MADRS; (27)]; (c) Hamilton Rating Scale for Anxiety [HAM-A; (28)]; (d) Mania Rating Scale [MRS; (29)].

The rating scales were administered at baseline (T_0_), at the end of the second week (T_1_), and at the end of the cycle of stimulation (T_2_). The MRS was mainly used as a clinical tool to monitor possible switches to manic or hypomanic episodes.

In order to avoid interfering factors, pharmacotherapy was implemented and maintained during the whole treatment.

### Data Analysis

The iTBS protocol effectiveness was assessed (i) at the subject level, by qualitatively comparing the rating scale scores over time, (ii) at the group-level, by quantitatively comparing the rating scale scores at different time points. In (ii), for each rating scale, a model with time as within-subject factor and the intercept as between-subject factor was fitted to the multiple scale measurements per subject (response variables). The model was interrogated through a repeated-measures analysis of variance (rANOVA). In the event of significant effects of time on the rating scale scores (*p* < 0.05), *post-hoc* pairwise time comparisons were performed.

## Results

The sample demographic and longitudinal clinical characteristics are reported in Table 1. Our group of patients was composed of four women and two men. Four patients were affected by MDD, two by BD II, with a mean age of 58.3 years and a standard deviation of 10.06 years. Two patients were taking mood stabilizing drugs (lithium or lamotrigine), five patients an atypical antipsychotic (quetiapine), two patients a second antidepressant, and two patients benzodiazepines.

**Table 1:** Demographic, clinical, and pharmacological variables of each subject.

	**Demographics**	**Clinical data**	**Current therapy**	**T0**	**T1**	**T2**
Subject 1	Age: 46 Sex: male	Diagnosis: unipolar TRD Duration of illness: 4 years Duration of last episode: 48 months	Clomipramine Bupropion	HAM-D: 16 MADRS: 12 HAM-A: 18 MRS: 0	HAM-D: 14 MADRS: 8 HAM-A: 12 MRS: 0	HAM-D: 17 MADRS: 13 HAM-A: 14 MRS: 0
Subject 2	Age: 58 Sex: male	Diagnosis: unipolar TRD Duration of illness: 20 years Duration of last episode: 5 months	Quetiapine Paroxetine Mirtazapine	HAM-D: 19 MADRS: 27 HAM-A: 19 MRS: 0	HAM-D: 20 MADRS: 25 HAM-A: 19 MRS: 0	HAM-D: 18 MADRS: 27 HAM-A: 18 MRS: 0
Subject 3	Age: 44 Sex: female	Diagnosis: TRD in BD type II Duration of illness: 24 years Duration of last episode: 4 months	Lamotrigine Quetiapine Duloxetine	HAM-D: 12 MADRS: 25 HAM-A: 13 MRS: 0	HAM-D: 12 MADRS: 23 HAM-A: 13 MRS: 0	HAM-D: 10 MADRS: 23 HAM-A: 12 MRS: 0
Subject 4	Age: 65 Sex: female	Diagnosis: TRD in BD type II Duration of illness: 10 years Duration of last episode: 3 months	Quetiapine Venlafaxine	HAM-D: 13 MADRS: 24 HAM-A: 14 MRS: 0	HAM-D: 18 MADRS: 25 HAM-A: 13 MRS: 0	HAM-D: 13 MADRS: 23 HAM-A: 12 MRS: 0
Subject 5	Age: 67 Sex: female	Diagnosis: unipolar TRD Duration of illness: 29 years Duration of last episode: 8 months	Quetiapine Clomipramine	HAM-D: 14 MADRS: 25 HAM-A: 8 MRS: 0	HAM-D: 15 MADRS: 28 HAM-A: 15 MRS: 0	HAM-D: 15 MADRS: 26 HAM-A: 12 MRS: 0
Subject 6	Age: 43 Sex: female	Diagnosis: unipolar TRD Duration of illness: 23 years Duration of last episode: 5 months	Lithium Quetiapine Clomipramine	HAM-D: 21 MADRS: 39 HAM-A: 10 MRS: 0	HAM-D: 19 MADRS: 34 HAM-A: 16 MRS: 0	HAM-D: 23 MADRS: 34 HAM-A: 15 MRS: 0

*TRBD, treatment-resistant bipolar depression; BD, Bipolar Disorder; HAM-D, Hamilton Depression Rating Scale; MADRS, Montgomery-Asberg Depression Rating Scale; HAM-A, Hamilton Rating Scale for Anxiety; MRS, Mania Rating Scale*.

**Figure 1:**
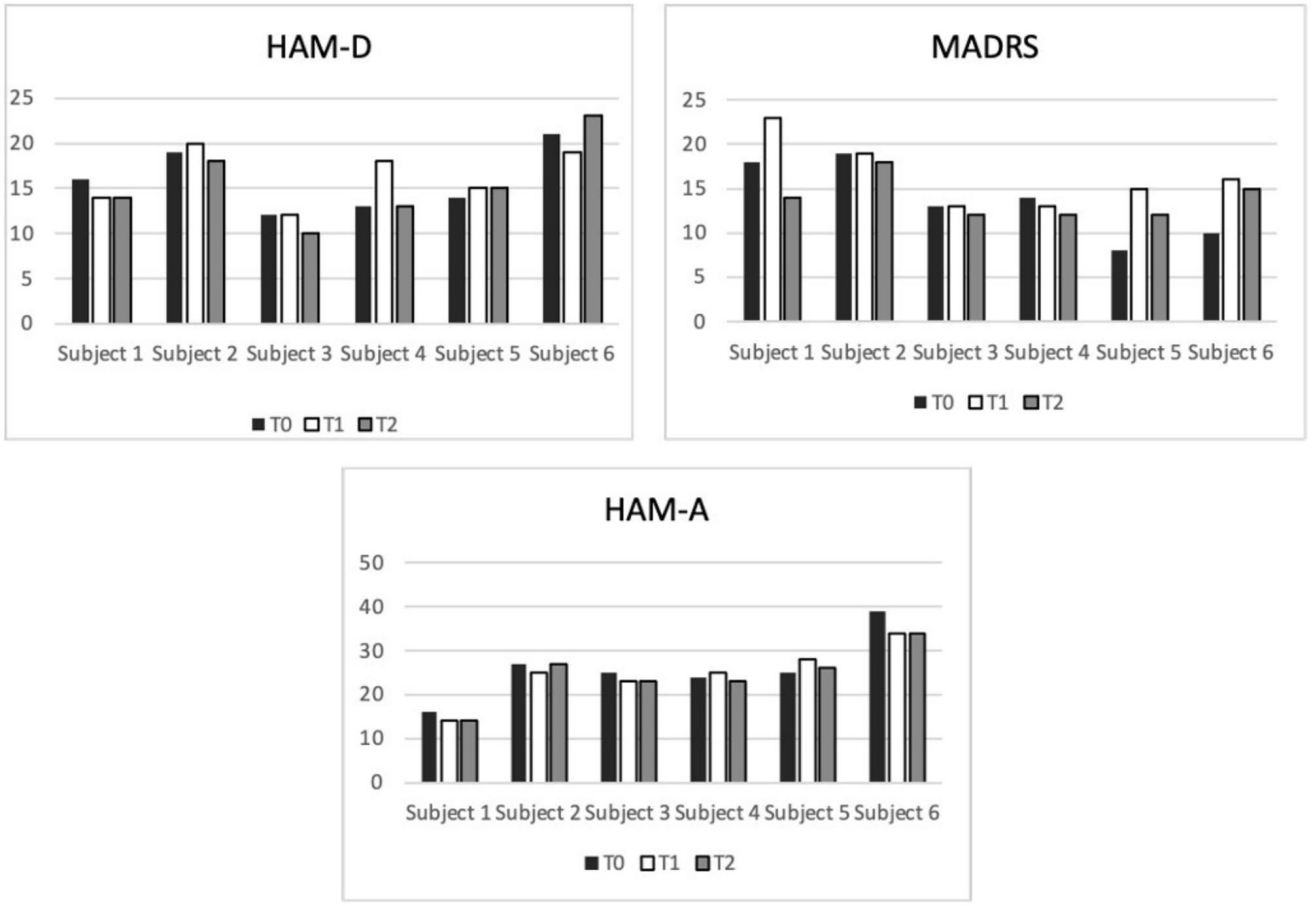
Bar diagrams showing subject-level depression and anxiety symptom scores before, during, and after iTBS protocol administration. On the X axis are displayed the subjects, on the Y the total scores. HAM-D, Hamilton Depressive Rating Scale; HAM-A, Hamilton Anxiety Rating Scale; MADRS, Montgomery-Asberg Depressive Rating Scale.

**Figure 2:**
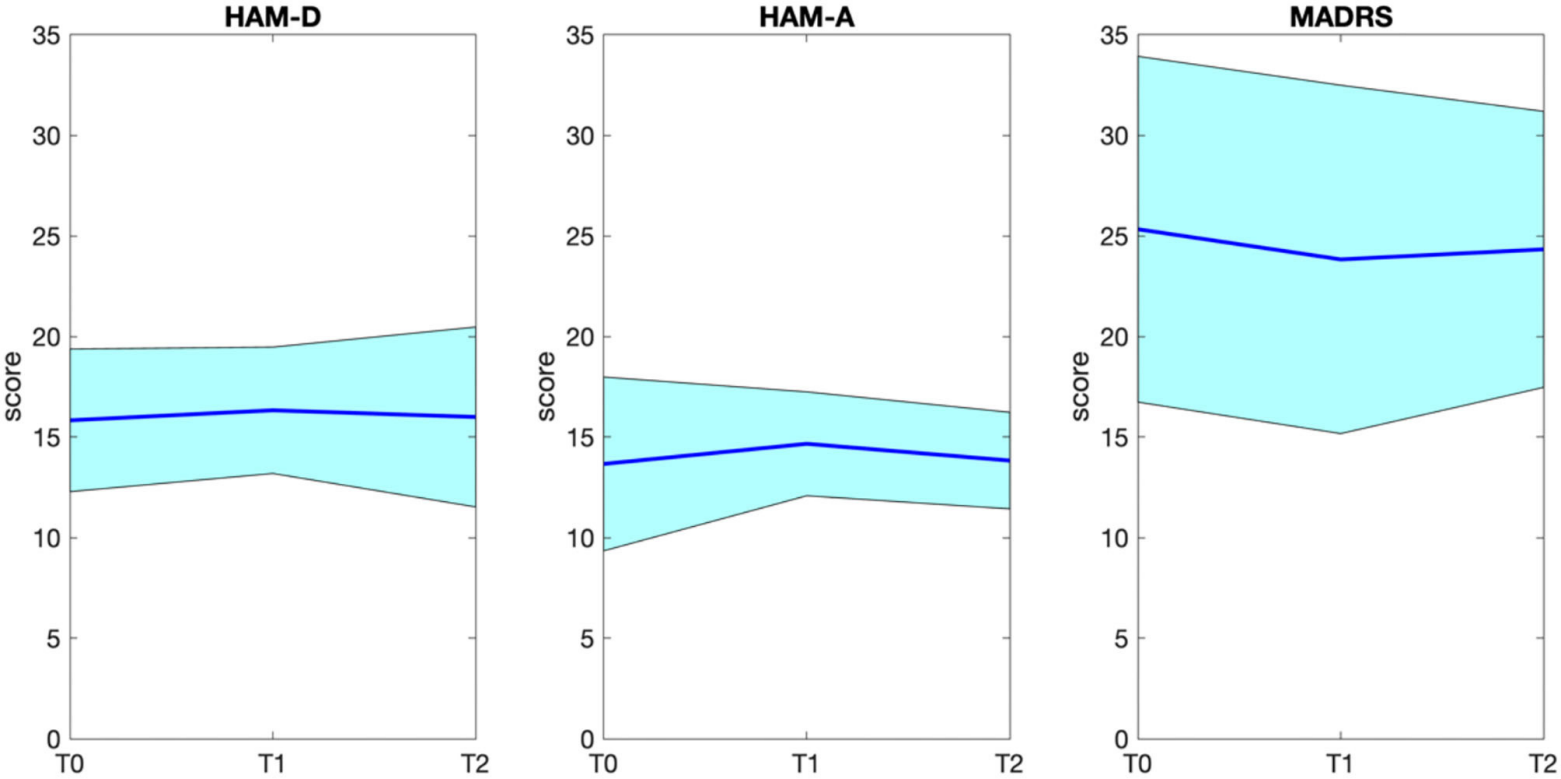
Group distribution (average ± standard deviation) of HAM-D, HAM-A, and MADRS scores before (T0), during (T1), and after (T2) iTBS protocol administration. HAM-D, Hamilton Depressive Rating Scale; HAM-A, Hamilton Anxiety Rating Scale; MADRS, Montgomery-Asberg Depressive Rating Scale.

The subject-level HAM-D, HAM-A, and MADRS scores from baseline to follow-up are shown in the bar diagrams of Figure 1. The group-level score distributions, shown in Figure 2, indicate a large inter-individual variability of the effects of accelerated iTBS on depressive and anxiety symptoms over time. The rANOVA statistics showed no significant effects of the time factor on the rating scale scores (HAM-D: *F* = 0.115, *p* = 0.749, HAM-A: *F* = 0.270, *p* = 0.626, MADRS: *F* = 0.981, *p* = 0.367), therefore no *post-hoc* pairwise comparisons (T0 vs. T1, T0 vs. T2, etc.) were performed. From T0 to T2, a slight decrease in MADRS scores was observed, accompanied by very slight increases in HAM-A and HAM-D scores. No manic symptoms appeared during the entire protocol.

The MRS, as expected, was consistent with a total score of 0 from T0 to T2 in each patient.

## Discussion

In this preliminary case series, we explored the effect of accelerated iTBS in six patients affected by drug-resistant MDD or BD. A robust pattern of improvement in terms of depressive symptoms did not emerge. The small sample size could have hampered the detection of the putative antidepressant effects of our protocol. Besides, three “technical” reasons could be highlighted.

First, a parameter that might have negatively impacted is the burst frequency of iTBS protocol, which was set to 30 Hz due to technical limitations of our equipment. Although 30-Hz iTBS frequency has been previously reported to be effective in reducing depressive symptoms (30–33), it is possible that a frequency closer to 50 Hz might have enhanced the iTBS efficacy. However, to date, a direct comparison between different iTBS frequencies has not been performed yet; future studies are warranted to further elucidate this issue.

A second parameter that might have influenced our results is the duration of the interval between sessions (15 min in our protocol). Indeed, contrasting results are present in the literature. On one hand, it has been found that in an accelerated protocol with 15-min intervals, the cortical excitability significantly increased up to 60 min post-stimulation (34). On the other hand, it has been reported that time intervals longer than 15 min are necessary to induce long term potentiation (LTP) in low-threshold synapses (35). This is consistent with a previous study showing that intervals from 40 to 50 min are required for the process of protein synthesis underlying the LTP effects (36). Therefore, the 15-min pause between stimulation sessions we used, which was similar to an accelerated rTMS protocol demonstrated to be as effective as the standard rTMS protocol in a recent study (20), could have been suboptimal.

Third, the heterogeneity in treatment regimens across our patients might have also limited the identification of significant effects of accelerated iTBS on clinical symptomatology. However, after enrolment in the study protocol, the doses of this variety of pharmacological treatments were not modified, thus reducing their possible confounding effects.

In conclusion, although accelerated iTBS is a less time-consuming approach for TMS administration useful in a public healthcare setting where time and personnel resources are often limited, our preliminary results in a real-world population affected by TRD did not show significant benefits on patients' symptomatology. Further studies on wider samples and exploring the effects of different accelerated iTBS parameters are needed to make definite conclusions on such a new and unexplored topic.

### Limitations

Our paper shows limitations which must be taken into account when reading our preliminary research. First, the reduced number of patients, even if producing significant data, might have produced limited amount of information. Another important aspect which might have impacted on our results is the absence of neuronavigation, which usually provides a more accurate administration of TMS. Further studies are necessary to implement our protocol with a more wide sample size.

## Data Availability Statement

The raw data supporting the conclusions of this article will be made available by the authors, without undue reservation.

## Ethics Statement

Ethical review and approval was not required for the study on human participants in accordance with the local legislation and institutional requirements. The patients/participants provided their written informed consent to participate in this study.

## Author Contributions

FC, GS, and PB contributed equally to the manuscript ideation. DS in ideation and in the recruitment of patients, while LC helped in writing minor corrections and in minor recruitment details. EM and GD gave important theatrical support for the manuscript writing and for statistical data. All authors contributed to the article and approved the submitted version.

## Conflict of Interest

The authors declare that the research was conducted in the absence of any commercial or financial relationships that could be construed as a potential conflict of interest.

## Publisher's Note

All claims expressed in this article are solely those of the authors and do not necessarily represent those of their affiliated organizations, or those of the publisher, the editors and the reviewers. Any product that may be evaluated in this article, or claim that may be made by its manufacturer, is not guaranteed or endorsed by the publisher.

## References

[1] Salagre E Solé B Tomioka Y Fernandes BS Hidalgo-Mazzei D Garriga M. Treatment of neurocognitive symptoms in unipolar depression: a systematic review and future perspectives. J Affect Disord. (2017) 221:205–21. 10.1016/j.jad.2017.06.034 28651185

[2] Merikangas KR Jin R He JP Kessler RC Lee S Sampson NA. Prevalence and correlates of bipolar spectrum disorder in the world mental health survey initiative. Arch Gen Psychiatry. (2011) 68:241–51. 10.1001/archgenpsychiatry.2011.12 21383262 PMC3486639

[3] Miller S Dell'Osso B Ketter TA. The prevalence and burden of bipolar depression. J Affect Disord. (2014) 169:S3–11. 10.1016/S0165-0327(14)70003-5 25533912

[4] Fitzgerald PB Chen L Richardson K Daskalakis ZJ Hoy KE. A pilot investigation of an intensive theta burst stimulation protocol for patients with treatment resistant depression. Brain Stimul. (2020) 13:137–44. 10.1016/j.brs.2019.08.013 31477542

[5] McConnell VL Carter SL Patterson K. Major depressive disorder: treatment-resistant depression and augmentation of other medication classes. Medsurg Nursing. (2019) 28:251–6. 10.2147/ndt.s3369 18696279

[6] Fava M. Diagnosis and definition of treatment-resistant depression. Biol Psychiatry. (2003) 53:649–59. 10.1016/S0006-3223(03)00231-2 12706951

[7] Sienaert P Lambrichts L Dols A De Fruyt J. Evidence-based treatment strategies for treatment-resistant bipolar depression: a systematic review. Bipolar Disord. (2013) 15:61–9. 10.1111/bdi.12026 23190379

[8] Hidalgo-Mazzei D Berk M Cipriani A Cleare AJ Florio AD Dietch D. Treatment-resistant and multi-therapy-resistant criteria for bipolar depression: consensus definition. Br J Psychiatry. (2019) 214:27–35. 10.1192/bjp.2018.257 30520709 PMC7613090

[9] Anderson IM. We all know what we mean by treatment-resistant depression–don't we? Br J Psychiatry. (2018) 212:259–61. 10.1192/bjp.2018.56 29693539

[10] Valero-Cabré A Amenguala JL Stengela C Pascual-Leone A Coubard OA. Transcranial magnetic stimulation in basic and clinical neuroscience: a comprehensive review of fundamental principles and novel insights. Neurosci Biobehav Rev. (2017) 83:381–404. 10.1016/j.neubiorev.2017.10.006 29032089

[11] Hallett M. Transcranial magnetic stimulation: a primer. Neuron. (2007) 55:187–99. 10.1016/j.neuron.2007.06.026 17640522

[12] Oberman L Edwards D Eldaief M Pascual-Leone A. Safety of theta burst transcranial magnetic stimulation: a systematic review of the literature. J Clin Neurophysiol. (2011) 28:67–74. 10.1097/WNP.0b013e318205135f 21221011 PMC3260517

[13] Hill AJ. First occurrence of hippocampal spatial firing in a new environment. Exp Neurol. (1978) 62:282–97. 10.1016/0014-4886(78)90058-4 729680

[14] Larson J Wong D Lynch G. Patterned stimulation at the theta frequency is optimal for the induction of hippocampal long-term potentiation. Brain Res. (1986) 368:347–50. 10.1016/0006-8993(86)90579-2 3697730

[15] Staubli U Lynch G. Stable hippocampal long-term potentiation elicited by ‘theta'pattern stimulation. Brain Res. (1987) 435:227–34. 10.1016/0006-8993(87)91605-2 3427453

[16] Klimesch W Doppelmayr M. Theta band power in the human scalp EEG and the encoding of new information. Neuroreport. (1996) 7:1235–40. 10.1097/00001756-199605170-00002 8817539

[17] Rachid F. Repetitive transcranial magnetic stimulation and treatment-emergent mania and hypomania: a review of the literature. J Psychiatr Pract. (2017) 23:150–9. 10.1097/PRA.0000000000000219 28291043

[18] Blumberger DM Vila-Rodriguez F Thorpe KE Feffer K Noda Y Giacobbe P. Effectiveness of theta burst versus high-frequency repetitive transcranial magnetic stimulation in patients with depression (THREE-D): a randomised non-inferiority trial. Lancet. (2018) 391:1683–92. 10.1016/S0140-6736(18)30295-2 29726344

[19] O'Reardon JP Solvason HB Janicak PG Sampson S Isenberg KE Nahas Z. Efficacy and safety of transcranial magnetic stimulation in the acute treatment of major depression: a multisite randomized controlled trial. Biol Psychiatry. (2007) 62:1208–16. 10.1016/j.biopsych.2007.01.018 17573044

[20] Fitzgerald PB Hoy KE Elliot D McQueen RS Wambeek LE Daskalakis ZJ. Accelerated repetitive transcranial magnetic stimulation in the treatment of depression. Neuropsychopharmacology. (2018) 43:1565–72. 10.1038/s41386-018-0009-9 29467437 PMC5983543

[21] Sonmez AI Camsari DD Nandakumar AL Voort JLV Kung S Lewis CP. Accelerated TMS for depression: a systematic review and meta-analysis. Psychiatry Res. (2019) 273:770–81. 10.1016/j.psychres.2018.12.041 31207865 PMC6582998

[22] Chung SW Hoy KE Fitzgerald PB. Theta-burst stimulation: a new form of TMS treatment for depression? Depress Anxiety. (2015) 32:182–92. 10.1002/da.22335 25450537

[23] Cole EJ Stimpson KH Bentzley BS Gulser M Cherian K Tischler C. Stanford accelerated intelligent neuromodulation therapy for treatment-resistant depression. Am J Psychiatry. (2020) 177:716–26. 10.1176/appi.ajp.2019.19070720 32252538

[24] McClintock SM Reti IM Carpenter LL McDonald WM Dubin M Taylor SF. Consensus recommendations for the clinical application of repetitive transcranial magnetic stimulation (rTMS) in the treatment of depression. J Clin Psychiatry. (2018) 79:16cs10905. 10.4088/JCP.16cs10905 28541649 PMC5846193

[25] Rossi S Hallett M Rossini PM Pascual-Leone A Safety Safety of TMS Consensus Group. Safety, ethical considerations, and application guidelines for the use of transcranial magnetic stimulation in clinical practice and research. Clin Neurophysiol. (2009) 120:2008–39. 10.1016/j.clinph.2009.08.016 19833552 PMC3260536

[26] Hamilton M. A rating scale for depression. J Neurol Neurosurg Psychiatry. (1960) 23:56–62. 10.1136/jnnp.23.1.56 14399272 PMC495331

[27] Montgomery SA Asberg M. A new depression scale designed to be sensitive to change. Br J Psychiatry. (1979) 134:382–9. 10.1192/bjp.134.4.382 444788

[28] Hamilton M. The assessment of anxiety states by rating. Br J Med Psychol. (1959) 32:50–5. 10.1111/j.2044-8341.1959.tb00467.x 13638508

[29] Young RC Biggs JT Ziegler VE Meyer DA. A rating scale for mania: reliability, validity and sensitivity. Br J Psychiatry. (1978) 133:429–35. 10.1192/bjp.133.5.429 728692

[30] Nyffeler T Wurtz P Luscher HR Hess CW Senn W Pflugshaupt T. Repetitive TMS over the human oculomotor cortex: comparison of 1-Hz and theta burst stimulation. Neurosci Lett. (2006) 409:57–60. 10.1016/j.neulet.2006.09.011 17049743

[31] Nyffeler T Cazzoli D Wurtz P Luthi M von Wartburg R Chaves S. Neglect-like visual exploration behaviour after theta burst transcranial magnetic stimulation of the right posterior parietal cortex. Eur J Neurosci. (2008) 27:1809–13. 18371083 10.1111/j.1460-9568.2008.06154.x

[32] Pedapati EV Gilbert DL Horn PS Huddleston DA Laue CS Shahana N. Effect of 30 Hz theta burst transcranial magnetic stimulation on the primary motor cortex in children and adolescents. Front Hum Neurosci. (2015) 9:91. 10.3389/fnhum.2015.00091 25762919 PMC4340218

[33] Wu SW Shahana N Huddleston DA Gilbert DL. Effects of 30Hz θ burst transcranial magnetic stimulation on the primary motor cortex. J Neurosci Methods. (2012) 208:161–4. 10.1016/j.jneumeth.2012.05.014 22627376 PMC3398243

[34] Tse NY Goldsworthy MR Ridding MC Coxon JP Fitzgerald PB Fornito A. The effect of stimulation interval on plasticity following repeated blocks of intermittent theta burst stimulation. Sci Rep. (2018) 8:8526. 10.1038/s41598-018-26791-w 29867191 PMC5986739

[35] Thomson AC Kenis G Tielens S de Graaf TA Schuhmann T Rutten BPF. Transcranial magnetic stimulation-induced plasticity mechanisms: TMS-related gene expression and morphology changes in a human neuron-like cell model. Front Mol Neurosci. (2020) 13:528396. 10.3389/fnmol.2020.528396 33192288 PMC7604533

[36] Kramár EA Babayan AH Gavin CF Cox CD Jafari M Gall CM. Synaptic evidence for the efficacy of spaced learning. Proc Natl Acad Sci USA. (2012) 109:5121–6. 10.1073/pnas.1120700109 22411798 PMC3323981

